# Compensation for Reflectance Variation in Vessel Density Quantification by Optical Coherence Tomography Angiography

**DOI:** 10.1167/iovs.16-20080

**Published:** 2016-08-29

**Authors:** Simon S. Gao, Yali Jia, Liang Liu, Miao Zhang, Hana L. Takusagawa, John C. Morrison, David Huang

**Affiliations:** Casey Eye Institute Oregon Health & Science University, Portland, Oregon, United States

**Keywords:** optical coherence tomography, optical coherence tomography angiography, reflectance compensation, vessel density, retina

## Abstract

**Purpose:**

To compensate for reflectance variation when quantifying vessel density by optical coherence tomography angiography (OCTA).

**Methods:**

Healthy participants received 6×6-mm macular and 4.5×4.5-mm optic nerve head (ONH) angiography scans on a 70-kHz spectral-domain optical coherence tomography system. The split-spectrum amplitude-decorrelation angiography (SSADA) algorithm was used to compute the OCTA signal. Mean reflectance projection and maximum decorrelation projection were used to create en face OCT and OCTA images. Background OCTA noise in static tissue was evaluated in the foveal avascular zone (FAZ). Vessel density was calculated from en face retinal OCTA that was binarized according to a decorrelation threshold.

**Results:**

The average retinal decorrelation noise in the FAZ was linearly related to the average logarithmic-scale OCT reflectance signal. Based on this relationship, a reflectance-adjusted decorrelation threshold equation was developed to filter out 97.5% of background OCTA noise. A fixed threshold was also used for comparison. The superficial vascular complex vessel density in the macula and ONH were significantly correlated with reflectance signal strength index (SSI) using the fixed threshold. This correlation was removed by using the reflectance-adjusted threshold. Reflectance compensation reduced population variation in 25 healthy eyes from 8.5% to 4.8% (coefficient of variation) in the macula and from 6.7% to 5.4% in the peripapillary region. Within-visit repeatability also improved from 4.4% to 1.8% in the macula and from 3% to 1.7% in the peripapillary region.

**Conclusions:**

Compensating for reflectance variation resulted in more reliable vessel density quantification in OCTA.

A number of ocular diseases that result in vision loss are related to changes in the retinal vasculature. Traditionally, fluorescein and/or indocyanine green angiography has been used to assess these changes, but objective quantification can be challenging due to blurring by dye leakage and/or staining and variation in contrast with dye transit time. Optical coherence tomography angiography (OCTA) is an emerging technology that utilizes variation in the OCT signal on consecutive cross-sectional B-scans at the same location to contrast flowing red blood cells in the vessel lumen from static tissue.^1–5^ Because OCTA has consistently high contrast for capillary details and is not affected by leakage and staining, quantification may be more straightforward. We previously developed an efficient OCTA algorithm called split-spectrum amplitude-decorrelation angiography (SSADA)^6,7^ and showed that it could be used to visualize and quantify changes in the vascular networks of the eye.^8,9^

Vessel density quantification using OCTA typically involves a threshold to be set on the en face angiogram to separate real flow signal in blood vessels from noise, which could arise from bulk tissue motion or from within the OCT system.^10,11^ On macular angiograms, the threshold can be based on the average flow signal in a background/noise region, the foveal avascular zone (FAZ), which is known to be free of blood vessels in healthy eyes. When using a fixed threshold for all pixels of an angiogram, we observed that vessel density appeared to be lower in regions where the OCT reflectance signal was weaker due to vitreous opacity or pupil edge vignetting. Because previous studies have shown that quantification metrics could be tied to the OCT reflectance signal strength,^12–14^ we sought to investigate and correct for this. We analyzed the relationship between the log OCT reflectance signal and decorrelation at the FAZ. We then determined whether a reflectance-adjusted threshold for flow detection could improve the reliability of vessel density measurements compared to the previous method of using a fixed threshold.

## Methods

### Study Information

This observational study was performed at the Casey Eye Institute. The research protocols were approved by the Institutional Review Board at the Oregon Health & Science University and carried out in accordance with the tenets of the Declaration of Helsinki. Written informed consent was obtained from each participant.

Healthy volunteers were recruited for the study. The inclusion criteria for healthy eyes were as follows: no evidence of retinal pathology or glaucoma; intraocular pressure less than 21 mm Hg; no chronic or systemic corticosteroid use; best-corrected visual acuity less than 20/40; and refractive error between −7 and +3 diopters.

### Optical Coherence Tomography Angiography

Two angiography scans were performed on single eyes of all participants. A subset of participants received optic nerve head (ONH) scans. Angiography scans were performed on a 70-kHz spectral-domain OCT system (RTVue-XR Avanti) with the AngioVue OCTA software (Optovue, Fremont, CA, USA). The angiography scan protocol contained two raster scans covering a 6×6-mm area for macular scans or 4.5×4.5-mm area for ONH scans. Each scan was composed of 304×304×2 A-scans acquired in less than 3 seconds. The fast scanning direction was in the horizontal direction for the first raster scan and in the vertical direction for the second. The SSADA algorithm was applied to detect flow by calculating the decorrelation of the OCT reflectance signal between the two consecutive B-scans at the same location.^6,7^ The raster scans were then registered and merged through an orthogonal registration algorithm to form a single set of OCT and OCTA volumes.^15^ The scanning software also computed a signal strength index (SSI) value based on the volumetric OCT reflectance signal. Signal strength index has often been used as an indicator of scan quality,^16–18^ with higher values being better.

### Neutral Density Filter to Reduce OCT Reflectance

Multiple macular angiography scans were performed on five additional healthy participants (age < 40 years). Each consecutive scan was collected with an absorptive neutral density filter (NDF) of increasing optical density (NEK01; Thorlab, Newton, NJ, USA) positioned in front of the eye. Optical densities ranging from 0.1 to 0.6 were used. Scans performed with higher optical density filters had lower SSI. These data were used to assess reflectance attenuation.

### Segmentation and En Face Presentation

For each angiography scan, the RTVue-XR output a registered, volumetric log reflectance amplitude volume (OCT) and decorrelation volume (OCTA). Because SSADA involves splitting the OCT interferogram, which reduces the axial resolution, the OCTA data have 160 voxels in each axial line (depth dimension). This was interpolated to 640 voxels to match the structural OCT data. In this manuscript, reflectance always refers to structural OCT information, which is log reflectance amplitude. Structural OCT was used for semiautomated segmentation of the inner limiting membrane (ILM), outer boundary of the inner plexiform layer (IPL), and inner/outer segment (IS/OS).^19^ To generate en face OCT and OCTA images, the average reflectance (mean projection) and maximum decorrelation (maximum projection) value for each transverse position within the segmented depth range was determined. Mean reflectance projection was used to provide en face images representative of all tissue in the slab. If maximum reflectance projection had been used, the image would have been dominated by a small number of intensely reflective voxels, and information on the beam coupling effect on average tissue reflectance would have been obscured. On the other hand, maximum decorrelation projection was used to accentuate flow signal in blood vessels that occupy only a small fraction of the tissue volume, without clutter or bias from the background static tissue, which occupy a much larger fraction of the slab volume.

### Data Analysis

Linear regression was used to assess the relationship between reflectance and decorrelation at the FAZ and between SSI and vessel density (Excel; Microsoft, Redmond, WA, USA). Vessel density was calculated from the en face decorrelation image (or angiogram) as previously described^10^ in custom software written in Matlab 2014a (MathWorks, Natick, MA, USA). A decorrelation threshold was used to separate vasculature from background noise. The pixels above the threshold divided by the total pixels in the region of interest was the vessel density. To compare vessel density calculations with and without compensating for reflectance, the average and population variation were reported. Coefficient of variation (CV) was used to assess population variation and within-visit repeatability (Excel).

## Results

Twenty-five healthy participants were included in this study. The age of the participants was 65 ± 9 years (average ± standard deviation; range, 43–80).

### Analysis of Reflectance and Decorrelation at the FAZ

**Figure 1:**
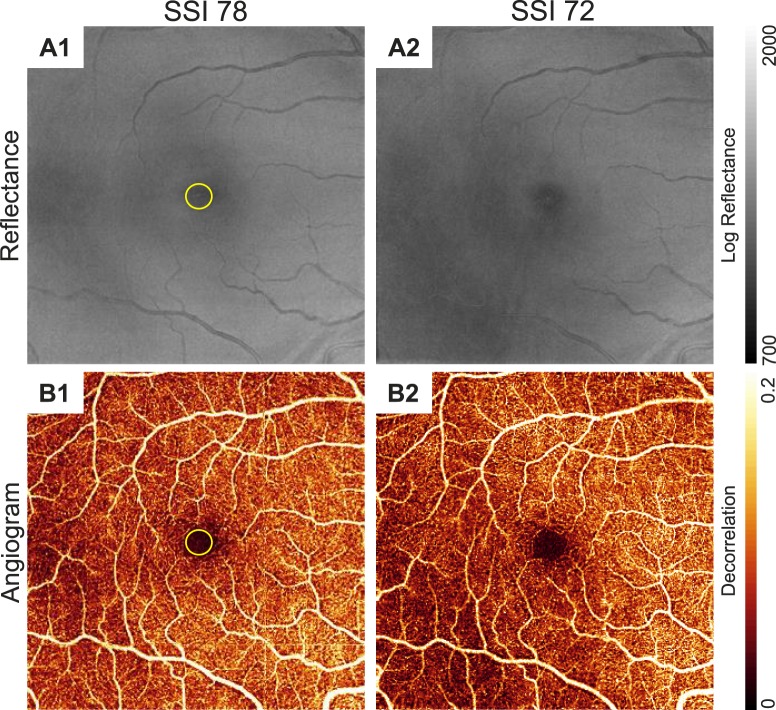
En face OCT reflectance images (**A1**, **A2**) and respective angiograms (**B1**, **B2**) showed variation at different signal strength index (SSI) values. Reflectance images were the maximum projection between the inner limiting membrane and outer boundary of the inner/outer segment. Angiograms were the maximum decorrelation projection between the same boundaries. The *yellow circle* identifies the foveal avascular zone. **A1**, **B1** and **A2**, **B2** were from the same participant; note the regional differences between **A1**, **B1** and **A2**, **B2** despite being from the same eye. *Scale bars on the right* show the range of log reflectance values on an arbitrary scale determined by the RTVue-XR and decorrelation flow signal.

We generated en face OCT reflectance images and angiograms by projecting the mean reflectance and maximum decorrelation, respectively, between the ILM and the outer boundary of IS/OS. Regional changes in reflectance within each image were observed (Fig. 1). The flow signal had matching regional variation with lower decorrelation values in regions with lower reflectance.

**Figure 2:**
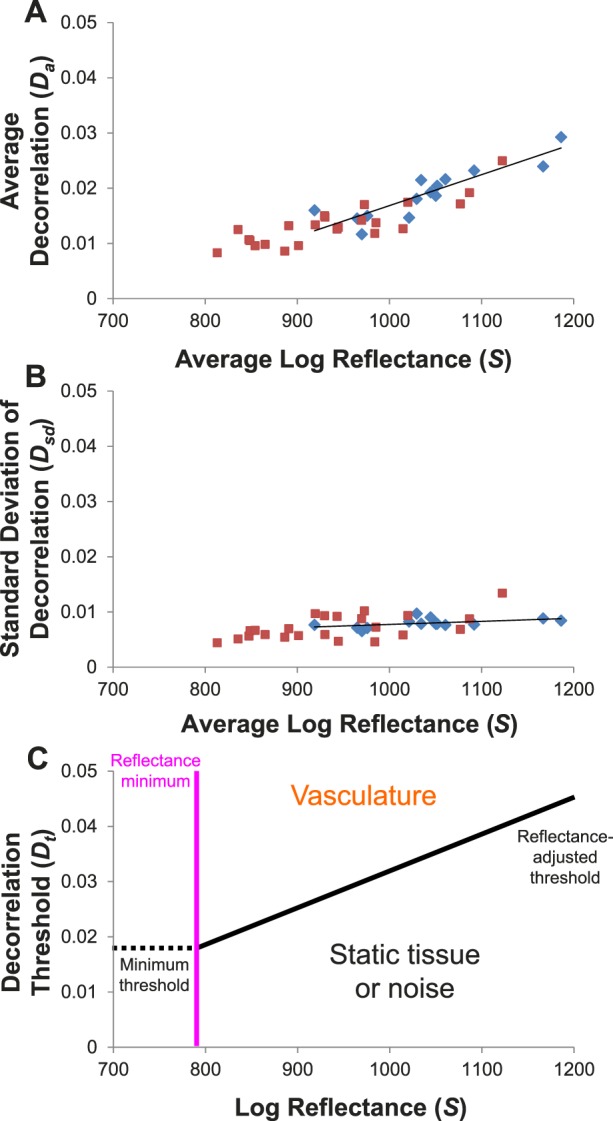
Analysis of OCT reflectance and decorrelation in the foveal avascular zone (FAZ) on en face images showed a positive linear relationship. Data in *blue* were from two scans of eight participants. Data in *red* were from five participants who received multiple scans with neutral density filters (NDFs) of varying optical densities. (**A**) Average FAZ decorrelation plotted against average log reflectance from each scan. (**B**) The standard deviation (SD) of the decorrelation plotted against average log reflectance. *Solid lines* show the linear fit of the data from the eight participants (*blue diamonds*), not including any of the data where NDFs were used (*red squares*). Signal attenuation by NDFs (*red squares*) followed the trend line established by interparticipant variation. This suggests that the interparticipant variation in average FAZ decorrelation is due to beam attenuation in ocular media or other factors that affect beam coupling. (**C**) The fits of the data were used to generate a reflectance-adjusted decorrelation threshold equation (Equation 5). Values above the threshold (*black line*) would be considered vasculature while values below would be static tissue. Values to the *left* of the reflectance minimum (*pink vertical line*) cannot be reliably classified because some flow signal would fall below the minimum threshold (*dotted horizontal line*).

To determine the relationship between OCT reflectance and background decorrelation noise in static tissue, we looked at the FAZ, which is known to be free of blood vessels. Data from 8 of the first 10 participants were used; 2 participants were not included due to the small size of their FAZ or motion-induced line artifacts within the FAZ. The SSI of the scans ranged from 61 to 78. The FAZ was selected from the en face OCTA. The hyperreflective foveal reflex was identified from en face structural OCT and removed from the analysis to avoid the associated decorrelation artifacts. Analysis of the average signal at the FAZ showed a positive linear relationship between OCTA decorrelation and log-scale OCT reflectance (Fig. 2). The linear fit of the average decorrelation *D_a_* to average log reflectance (Fig. 2A) was

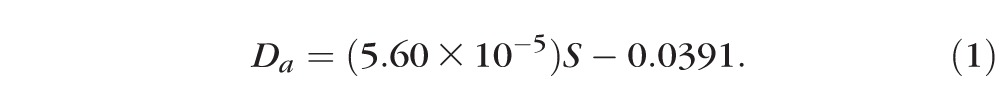



The decorrelation values of the FAZ pixels within the en face angiogram could be approximated with a normal distribution (Supplementary Fig..). The linear fit of the standard deviation of the decorrelation *D_sd_* to average log reflectance (Fig. 2B) was

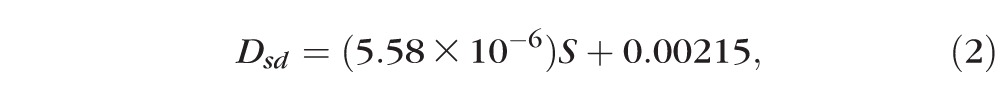
where *S* is the log amplitude reflectance signal from the RTVue-XR. The relationship between the reflectance amplitude *R* and the RTVue-XR signal *S*, based on optical bench calibration, was found to be^20^

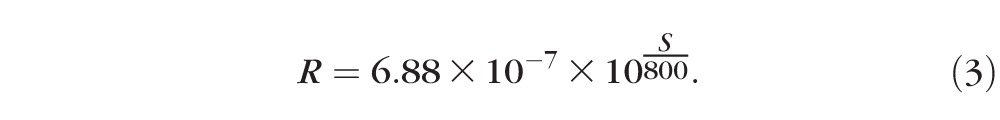



Equations 1 and 2 were used to generate the reflectance-adjusted threshold equation

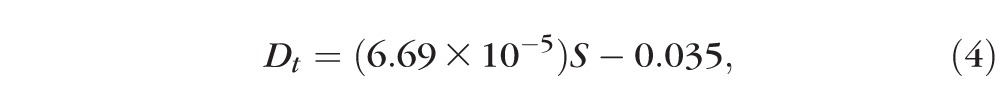
where *D_t_* is the decorrelation threshold. Equation 4 was set at the average (Equation 1) plus 1.96 times the standard deviation (Equation 2), representing the 97.5 percentile point of a normal distribution. Alternatively, a fixed threshold using the same data and 97.5 percentile criteria gave a decorrelation of 0.0347.

Before the reflectance-adjusted threshold equation could be used, two additional modifications were necessary. Based on our NDF data, we found that signal attenuation by NDFs approximated the effect of interindividual variation (Figs. 2A, 2B). If we take the reflectance of a tissue region as measured by the OCT signal amplitude to include effects from both beam coupling and intrinsic tissue reflectivity, where beam coupling is the efficiency with which light reflected from tissue is coupled into the OCT system and would be affected by focus, aberrations, and attenuation from ocular media, this suggested that the dependence of background decorrelation on log reflectance was likely due to beam coupling rather than differences in intrinsic retinal tissue reflectivity. Therefore, we introduced a *S*_offset_ term to remove the tissue reflectivity differences between the reference tissue slab used for compensation and the FAZ used in calibration to recover the information on beam coupling. Furthermore, *D_a_* appears to reach a minimum below an *S* of ∼900. To prevent the decorrelation signal in extremely low reflectance regions from being counted as vasculature, we chose to set a minimum reflectance threshold (Fig. 2C). We used the mean plus 1.28 times the standard deviation, 90% percentile point, of the nine NDF data points below an *S* of ∼900 to determine a minimum *D_t_*. Back calculation using Equation 4 gives a reflectance minimum (*S* – *S*_offset_) of 787. Areas that were below the reflectance minimum were considered invalid pixels and not included in quantification. To reduce the number of pixels corresponding to large retinal vessels being considered invalid, a circular median filter with a radius of 8 pixels (diameter of 320 μm for 6×6-mm macular scans) was first used on the reflectance image. The final reflectance-adjusted threshold equation was then as follows:

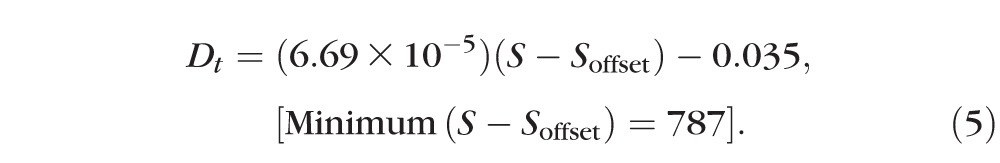



### Vessel Density With and Without Reflectance Compensation

**Figure 3:**
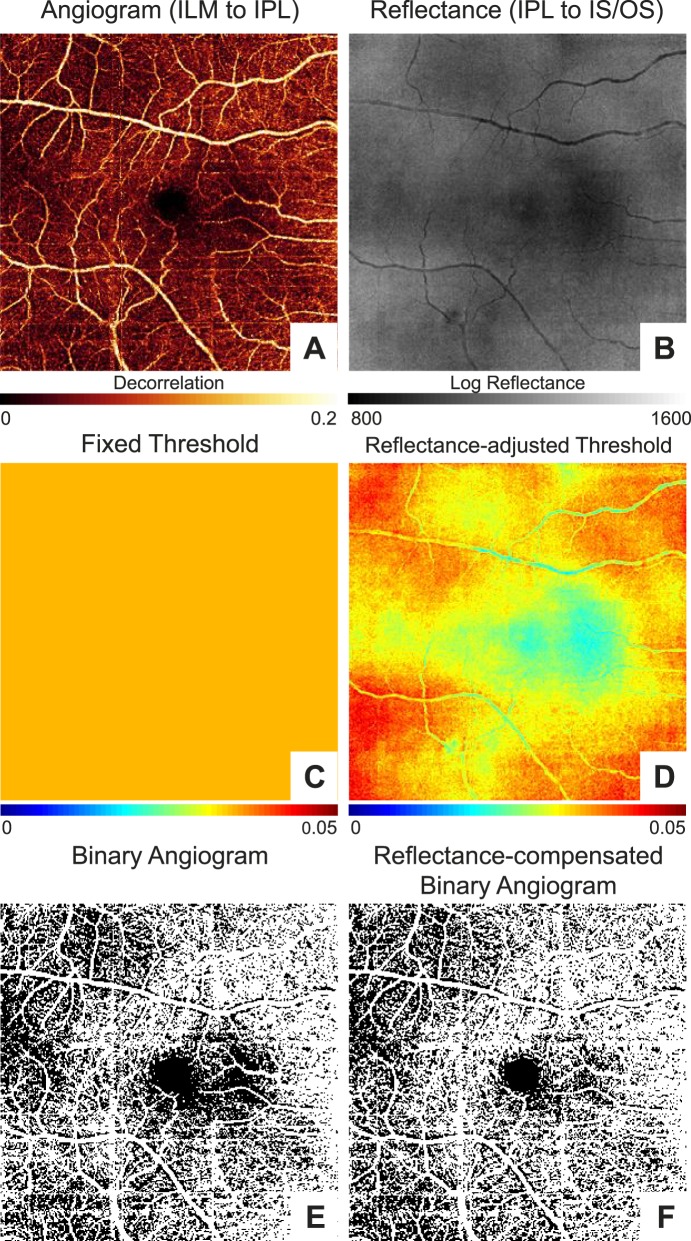
An example of the macula in a right eye to show the improvement in vessel density uniformity using the reflectance-adjusted threshold compared to a fixed threshold. (**A**) En face angiogram generated by maximum flow projection in the superficial vascular complex defined as between the inner limiting membrane (ILM) and outer boundary of the inner plexiform layer (IPL). (**B**) En face reflectance image generated by the mean log reflectance of the slab between the outer boundaries of IPL and hyperreflective inner/outer segment (IS/OS) junction band. (**C**) Threshold map with a fixed value of 0.0347. (**D**) Reflectance-adjusted threshold map derived from the reflectance map shown in (**B**) and Equation 5. (**E**) Binarized image of (**A**) based on a fixed threshold for all pixels as shown in (**C**). (**F**) Binarized image of (**A**) based on the reflectance-adjusted threshold map in (**D**). Note the apparent nonperfusion defect in the region immediately to the *right* (nasal) of the fovea in (**E**) that was improved in (**F**), which compensated for the low reflectance in that area (**B**, **D**) likely due to a vitreous opacity. Vessel density is higher on the nasal (disc) side of the image; this is a normal pattern associated with the thicker nerve fiber layer around the optic disc.

Next, we tested to see the effect of compensating for reflectance in our set of macular scans from the 25 healthy participants. En face angiograms of the superficial vascular complex (including both the radial peripapillary capillary plexus and the superficial vascular plexus)^20^ of the retina were generated by projecting the maximum decorrelation between ILM and the outer boundary of the IPL. Vessel density outside of a 0.6-mm-diameter disc centered at the FAZ was quantified using a fixed threshold of 0.0347 for all pixels on the angiogram and with thresholds derived from Equation 5. The reflectance values used to calculate the reflectance-adjusted threshold were based on the mean projection between the outer boundaries of the IPL and IS/OS. Instead of using the reflectance values directly, we first subtracted 64.4 from all values to account for the reflectance difference between the regions used for compensation and where the reflectance-adjusted threshold equation was derived. The 64.4 offset (*S*_offset_) was the average reflectance between the outer boundaries of the IPL and IS/OS outside of the 0.6-mm-diameter disc centered at the FAZ (1131.5) minus the average reflectance within a 0.3-mm-diameter disc centered at the FAZ (1067.1) in the 16 scans from 8 participants used to derive Equations 1 and 2. Figure 3 shows an example case in which regional variation in vessel density near the macula was reduced using the reflectance-adjusted threshold.

**Figure 4:**
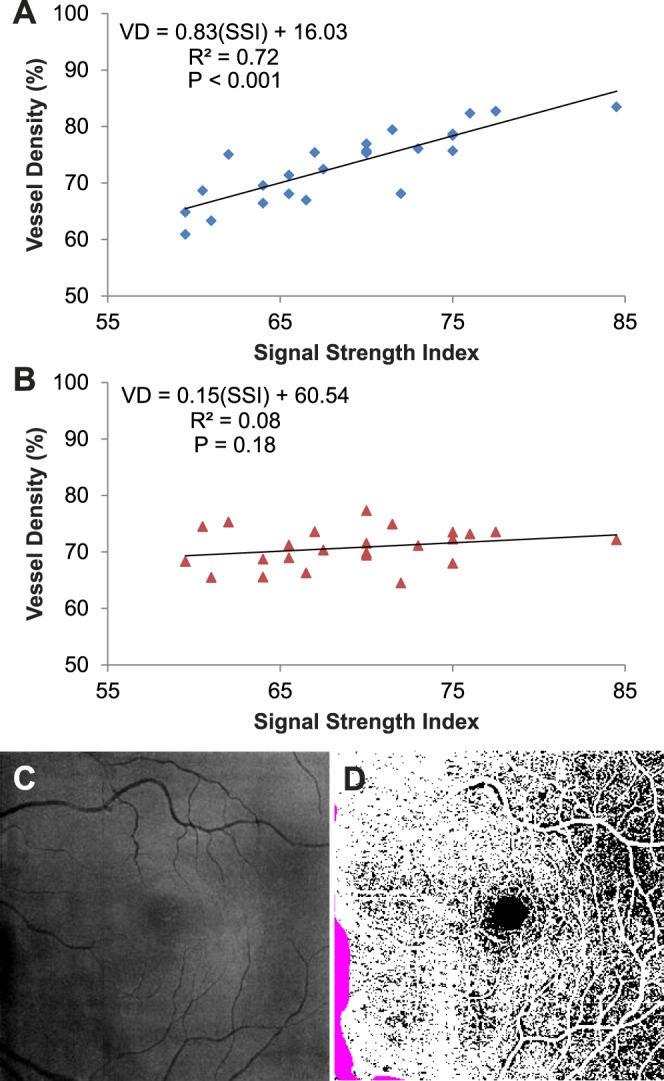
The linear relationship between signal strength index (SSI) and vessel density (VD) at the macula determined using a fixed threshold (**A**) was reduced with the reflectance-adjusted threshold (**B**). Each data point was the average of the two scans from a participant. Linear regression was used to fit the data. The fit equation, *R*^2^, and *P* values are shown. (**C**) En face reflectance image (IPL to IS/OS) of the scan with the highest percentage of invalid pixels (2.3%). (**D**) The corresponding reflectance-compensated binary angiogram with the invalid pixels in *purple*. As noted in Figure 3, vessel density is higher on the nasal (disc) side of the image.

When using a fixed threshold, we observed the expected positive relationship between SSI and vessel density (Fig. 4A). Linear regression gave a fit with a slope of 0.83, *R*^2^ of 0.72, and *P* < 0.001. If the reflectance-adjusted threshold was used instead, the relationship was reduced (Fig. 4B). The linear fit had a shallower slope of 0.15, *R*^2^ of 0.08, and *P* = 0.18. The majority of the cases had less than 1% of the pixels identified as invalid. In the scan with the highest percentage of invalid pixels, this was 2.3% (Figs. 4C, 4D). The Table shows the average vessel density values, population variation, and within-visit repeatability of the data in Figure 4.

**Table.**
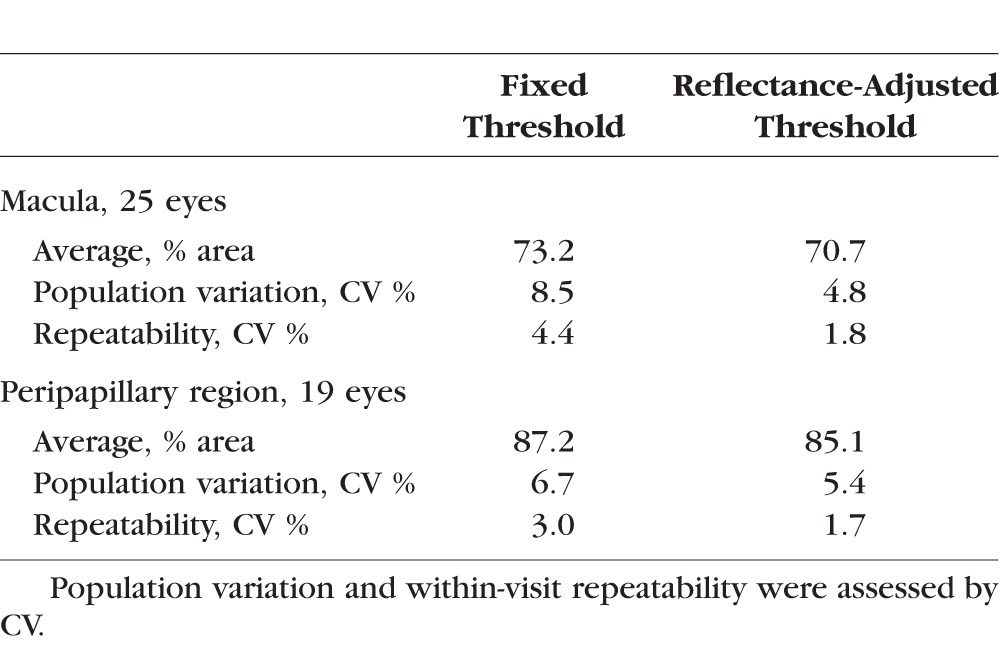
Vessel Density in the Retinal Superficial Vascular Complex in Healthy Participants

**Figure 5:**
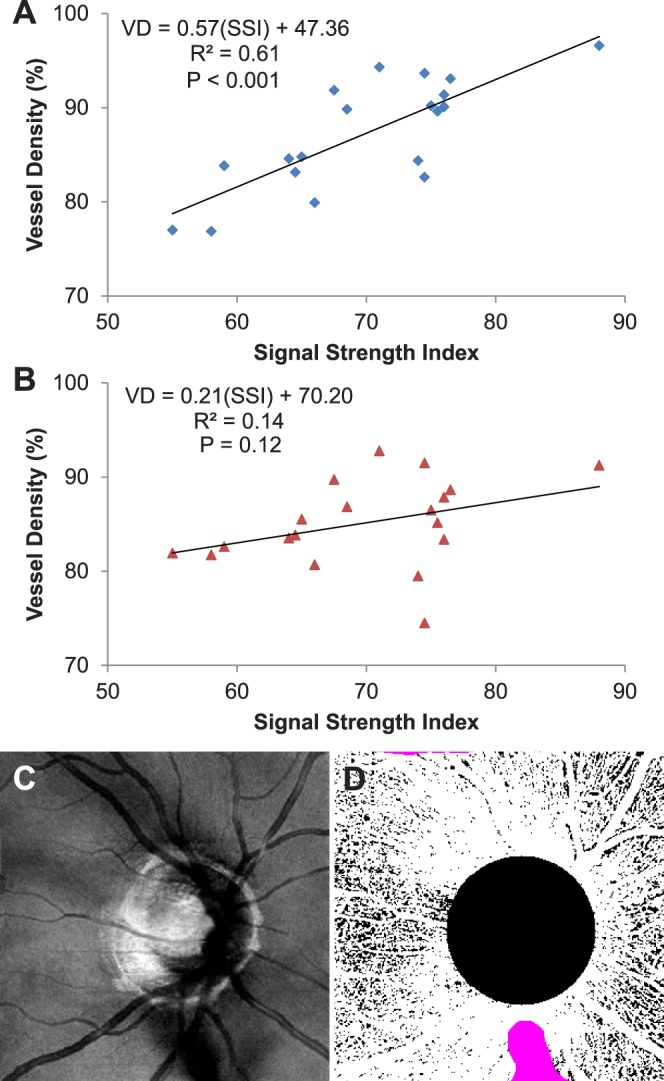
The linear relationship between signal strength index (SSI) and vessel density (VD) at the peripapillary region determined using a fixed threshold (**A**) was reduced with the reflectance-adjusted threshold (**B**). Each data point was the average of the two scans from a participant. Linear regression was used to fit the data. The fit equation, *R*^2^, and *P* values are shown. (**C**) En face reflectance image (IPL to IS/OS) of the scan with the highest percentage of invalid pixels (2.9%). (**D**) The corresponding reflectance-compensated binary angiogram with the invalid pixels in *purple*.

Finally, we assessed the effect of compensating for reflectance in our set of ONH scans. Of the 25 participants, 19 had two volumetric scans. The compensation approach used for the macular scans was directly translated. The only modifications were as follows: (1) The region of interest was the peripapillary retina, defined as the region outside of a 2-mm-diameter disc centered on the ONH; (2) the *S*_offset_ was 73.5, based on the average reflectance of the peripapillary region (1140.6) minus the average reflectance within the FAZ defined previously; and (3) the size of the circular median filter was increased to 17 pixels (diameter of 503 μm for 4.5×4.5-mm ONH scans) to avoid larger peripapillary blood vessels being counted as invalid regions. As was the case for macular scans, using the reflectance-adjusted threshold reduced SSI and vessel density correlation in the peripapillary region (Fig. 5; slope = 0.57, *R*^2^ = 0.61, *P* < 0.001 to slope = 0.21, *R*^2^ = 0.14, *P* = 0.12). The average vessel density values, population variation, and within-visit repeatability of the data in Figure 5 are shown in the Table.

## Discussion

Optical coherence tomography angiography offers new opportunities for diagnosing vascular diseases^21,22^ and tracking disease progression or response to treatment.^23^ For example, eyes with diabetic retinopathy^24^ and glaucoma^9^ show a significant decrease in vessel density compared to controls. Accurate and repeatable quantification metrics such as vessel density are essential. However, previous studies noted that quantitative metrics from OCT were correlated to reflectance signal strength.^12–14^ Here, we analyzed the background decorrelation in static tissue and used the average reflectance signal over several anatomic layers to obtain a measure of OCT beam coupling, which was confirmed by using NDFs. Beam coupling is the efficiency with which tissue backscattering is coupled into the sample arm optical fiber of the OCT system. It is affected by beam defocus, scattering, blocking, and absorption due to ocular refractive error, aberration, and media opacity. Information from the FAZ was used to generate an equation to calculate the reflectance-adjusted threshold for flow detection. We showed that compensating for reflectance variation reduced the dependence on reflectance signal strength and resulted in vessel density quantification of the superficial vascular complex that was more reliable, with improved population variation and within-visit repeatability.

Although our study was focused on a single OCTA algorithm, our general approach of reflectance compensation should be applicable to other OCTA implementations. The empirically derived coefficients used in the reflectance compensation formula would need to be derived using data obtained with the other algorithms, however, as the flow signal values would be different. Furthermore, we found that the distribution of decorrelation and reflectance values in en face images of the FAZ and vascular retina were minimally affected by the size of the OCTA scans investigated (4.5×4.5 and 6×6 mm). Therefore, within this range of scan sizes, the method should also be directly applicable. A scan size–dependent recalibration could produce slight adjustments to the formula coefficients.

This trend of increased decorrelation background noise with increased reflectance is counterintuitive. One would think that background noise would increase with insufficient reflectance signal. However, in the SSADA algorithm, a floor value is used to prevent very low reflectance amplitudes near zero from creating high decorrelation values. This low-signal floor filters out the background noise associated with low reflectance signal and prevents low reflectance layers (e.g., vitreous or ONL) from having high decorrelation signal (flow noise). Depending on how this filter is applied to the spectrally split images in SSADA, it may contribute to why both real flow signal and decorrelation noise increased with reflectance amplitude.

An important step in implementing our approach for discriminating vessel pixels from static tissue pixels on en face angiograms is choosing what reflectance values to use in Equation 5. When assessing the flow information between the ILM and outer boundary of IPL, we would ideally use the reflectance information from the same slab when determining the reflectance-adjusted threshold. However, the NFL thins more peripherally, which would change the reflectivity of the slab transversely and induce an artifactual gradient in threshold values. Thus, we chose to use reflectance values from the en face image generated by the projection between the outer boundaries of IPL and IS/OS. Using these values directly, however, resulted in thresholds that were too high. Because we derived the reflectance-adjusted threshold equation from the FAZ, composed largely of outer nuclear layer, which has low reflectivity, we needed an offset to account for reflectivity differences between the parafoveal/perifoveal and peripapillary regions from the FAZ.

A linear reflectance-adjusted threshold equation was used in our implementation. However, the NDF data showed that the average decorrelation in the FAZ reached a floor. Thus, we set a minimum (reflectance) threshold for which pixels would be considered in the vessel density quantification to prevent the decorrelation signal in extremely low reflectance regions from being counted as vasculature. This serves as a regional quality control metric such that if invalid pixels in a region reach greater than a certain percentage (e.g., 10%), those data can be discarded. To avoid invalidating pixels due to local tissue reflectance variation (e.g., low reflectance beneath large retinal vessels), a circular median filter was applied to the reflectance image to filter out focal variation while retaining information on OCT beam coupling. We found that setting the filter size to be roughly two to three times the diameter of the largest expected vessel was adequate.

Because we focused only on the superficial vascular complex vessel density in healthy eyes, additional studies are needed to investigate using the reflectance-adjusted threshold for assessment of vessel density in other layers (e.g., deeper retinal plexuses and choriocapillaris) and in disease. While the general approach should be transferrable, modifications with regard to which layers are used for compensation may be needed. For the choriocapillaris, the layer used for compensation will likely need to be from below Bruch's membrane to be able to account for shadowing due to the retinal pigment epithelium and drusen. Modification of the reference reflectance measurement may be needed in specific diseases. For example, cystic spaces appear as low-reflectance regions on en face structural OCT that could lead to artifactually lower thresholds. This local variation in tissue reflectivity may already be adequately addressed by our use of transverse median filtering. Whether additional modifications are needed will need to be studied in each type of disease. Beyond vessel density, reflectance compensation should be investigated for other metrics used to quantify OCTA data. For example, nonperfusion or capillary dropout area has been shown to be a sensitive biomarker for detecting diabetic retinopathy.^24^ Reflectance compensation could improve visualization and quantification of nonperfusion area such that regions similar to what is seen in Figure 3 are not incorrectly counted. Finally, we would like to note that reflectance compensation of decorrelation values in the vessels for the computation of flow index or other parameters that relate to flow velocity is another interesting avenue for further investigation.

Without compensation, signal strength variation due to refractive error, cataract, and other media opacities could lead to artifactual identification of capillary dropout or regions with low vessel density measurements. This has implications for OCTA assessment of a wide variety of diseases, including glaucoma^9^ and diabetic retinopathy.^24,25^ Our results show that a reflectance compensation approach could largely resolve this problem, and further investigations in clinical studies of disease states are warranted.

## Supplementary Material

Supplement 1

Supplement 2
