# Supplementary figures and images for: Compensation for Reflectance Variation in Vessel Density Quantification by Optical Coherence Tomography Angiography
Source: Invest Ophthalmol Vis Sci. 2016 Aug 29;57(10):4485–92. doi: 10.1167/iovs.16-20080 (PMC5015963; doi:10.1167/iovs.16-20080)

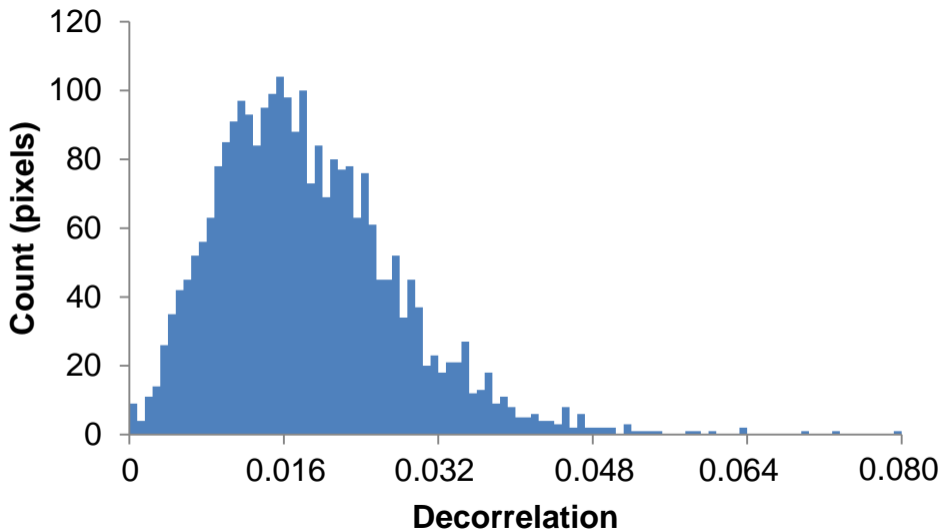

Supplement: Supplement 1 [file i1552-5783-57-10-4485-s01.pdf]
