# Supplementary material for: Compensation for Reflectance Variation in Vessel Density Quantification by Optical Coherence Tomography Angiography
Source: Invest Ophthalmol Vis Sci. 2016 Aug 29;57(10):4485–92. doi: 10.1167/iovs.16-20080 (PMC5015963; doi:10.1167/iovs.16-20080)
Supplement: Supplement 2 [file i1552-5783-57-10-4485-s02.pdf]

SUPPLEMENTARY FIGURE LEGENDS

SUPPLEMENTARY FIGURE. Histogram of the decorrelation values of FAZ pixels within *en face* angiograms.
